# Limitations of Oral Mucosal Moisture Assessment as an Indicator of Intravascular Dehydration in Postoperative Cardiovascular Surgical Patients in the ICU: A Prospective Pilot Diagnostic Accuracy Study

**DOI:** 10.7759/cureus.100502

**Published:** 2025-12-31

**Authors:** Toshiharu Nakama, Takafumi Todaka, Chihaya Uehara, Takumi Furugen, Mitsunobu Toyosaki

**Affiliations:** 1 Adult Nursing, Faculty of Nursing, Daiichi University of Pharmacy, Fukuoka, JPN; 2 Intensive Care Unit, Yuuai Medical Center, Tomigusuku, JPN; 3 Dentistry, Yuuai Medical Center, Tomigusuku, JPN; 4 Clinical Engineering, Yuuai Medical Center, Tomigusuku, JPN

**Keywords:** diagnostic accuracy, hypovolemia, intravascular volume depletion, mucus, oral mucosal moisture, postoperative cardiac surgery, venous excess ultrasound

## Abstract

Background: Oral mucosal moisture (OMM) measured by using a portable device (Mucus^®^, LiFE Co., Ltd., Koshigaya, Saitama, Japan; approval No. 22200BZX00640000) quantifies tongue moisture, yet its diagnostic value for intravascular volume depletion (hypovolemia) in critically ill patients remains unclear.

Objectives: To evaluate the diagnostic accuracy of OMM for hypovolemia in postoperative cardiovascular surgical patients in the intensive care unit (ICU).

Methods: The present single-center, prospective diagnostic accuracy study (Japan, Nov 2024-Sep 2025) enrolled consecutive postoperative cardiovascular surgical adults (≥20 years). OMM (index test) was measured on postoperative day (POD) 2 or on POD 3, if the POD 2 data were unavailable. The reference standard was a composite requiring all of the following conditions: absence of venous congestion (venous excess ultrasound (VExUS) score = 0), at least one low-volume indicator (central venous pressure (CVP) <4 mmHg, small inferior vena cava (IVC) diameter, or positive collapsibility), and a positive left ventricular (LV) “kissing sign,” acquired within a two-hour window. The predefined tongue dryness cutoff (<27.9%) and a data-driven Youden threshold were compared. We estimated the sensitivity, specificity, predictive values, likelihood ratios, and area under the receiver-operating characteristic curve with confidence intervals (CIs). Multivariable logistic regression was performed after adjusting for age, Acute Physiology and Chronic Health Evaluation II (APACHE II) score, and body mass index. Separately, the correlations between OMM and CVP, IVC diameter, VExUS score, and intraoperative water balance were examined.

Results: Among the 85 eligible patients, 57 were enrolled (age, 66.9 ± 10.7 years; 70.2% were male). The hypovolemia prevalence was 12.3%. At the cutoff Youden index (≤26.1), sensitivity was 0.714 (95% CI 0.290-0.963); specificity, 0.640 (0.492-0.771); positive predictive value (PPV), 0.217 (0.075-0.437); negative predictive value (NPV), 0.941 (0.803-0.993); and AUC, 0.640 (0.476-0.804). At the predefined cutoff (<27.9%), sensitivity was 0.857 (0.421-0.996); specificity, 0.460 (0.318-0.607); PPV, 0.182 (0.070-0.355); and NPV, 0.958 (0.789-0.999). The diagnostic accuracy did not differ between the two thresholds (McNemar p > 0.99). OMM was not independently associated with hypovolemia and showed no significant correlations with CVP, IVC diameter, VExUS score, or intraoperative balance.

Conclusions: In this single-center postoperative cardiac ICU cohort study, OMM showed limited discrimination for hypovolemia and no clear association with intravascular volume. OMM is not supported as a stand-alone screening tool; any interpretation should be coupled with dynamic hemodynamic assessments.

## Introduction

Dehydration is common in clinical practice, requiring timely assessment. Oral dryness, long considered a classic sign, is now regarded as a supplementary rather than a primary diagnostic finding owing to concerns about its specificity, predictive accuracy, and subjectivity of its assessment (variability between observers and dependency on context) [1]. Nonetheless, device-based measurement of oral mucosal moisture (OMM) offers rapid and more objective quantification of oral surface hydration. However, whether OMM can reflect intravascular volume status remains uncertain, particularly in postoperative cardiovascular intensive care unit (ICU) patients. In this setting, OMM may be influenced by ICU-related factors that affect local oral hydration, while the early postoperative period often involves rapid hemodynamic changes and fluid redistribution, potentially creating a physiological mismatch between oral surface moisture and intravascular hypovolemia. Thus, in this population, superficial mucosal hydration may not reliably mirror effective circulating volume. The objective of this study was to prospectively evaluate whether OMM can serve as a diagnostic indicator of intravascular hypovolemia in postoperative cardiovascular surgery patients admitted to the ICU.

Devices quantifying tongue mucosal moisture (Mucus®, LiFE Co., Ltd., Koshigaya, Saitama, Japan; approval No. 22200BZX00640000) have been utilized in dentistry and elderly care [2-4], with diagnostic applications established for dry-mouth evaluations in dental hygiene departments [3] and long-term care facilities [4]. In critical care, to the best of our knowledge, published evidence is limited to a small, single-center emergency-room study (N = 30) reporting a negative correlation between dehydration level and OMM, r = −0.686 [2]. However, the dehydration grading utilized in the previous study was a composite of laboratory tests and clinician judgment, which could introduce subjective elements, representing a design limitation. Mucus quantifies superficial tongue mucosal moisture within seconds using a dielectric constant and shows good inter-operator reproducibility [5]. However, no unified cutoff currently exists for systemic/intravascular dehydration [2-5]; the reported thresholds (≥29.6% (normal), 28.0%-29.5% (borderline), and <27.9% (dry)) were developed for classifying dry mouth (xerostomia), rather than for evaluating systemic or intravascular volume status [3,5,6]. Traditionally, medical textbooks have referred to oral dryness as a typical sign of dehydration [1,7], and dehydration, defined as a deficit of free water in total body water, and volume depletion/hypovolemia, defined as a loss of extracellular volume, particularly the intravascular effective circulating volume, have often been conflated [7,8]. As such, OMM may primarily reflect mucosal surface hydration (extravascular), rather than intravascular volume, and, therefore, may fail to accurately detect relative intravascular dehydration. Hereafter, “hypovolemia” refers to intravascular volume depletion. This concern is particularly salient in ICU patients, in whom inflammatory responses and surgical invasion provoke capillary leak, shifting fluid extravascularly and causing tissue edema while the intravascular compartment becomes relatively depleted [9]. This physiology may accentuate the mismatch between the observed layer (oral mucosal surface) and the intended target (intravascular volume). This putative mismatch suggests a theoretical limitation of using OMM as a screening test for hypovolemia; however, it currently remains a hypothesis that has not been adequately investigated.

## Materials and methods

Study design and participants

**Figure 1:**
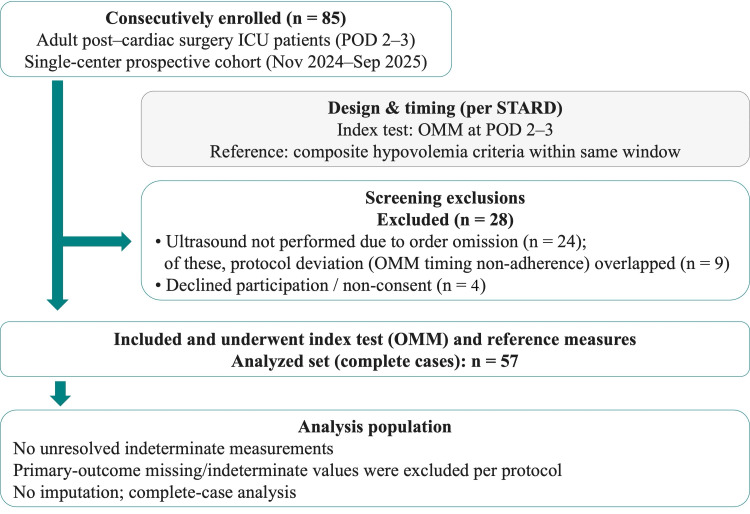
STARD participant flow diagram Overall, 57/85 (67.1%) of enrolled patients remained for the final analysis. OMM: oral mucosal moisture values; POD: postoperative day; ICU: intensive care unit; STARD: Standards for Reporting Diagnostic Accuracy Studies

The present investigation is a single-center prospective cohort study conducted at Yuuai Medical Center, an acute-care hospital in Tomigusuku, Okinawa, Japan. We utilized OMM as an adjunctive screening index, with measurements obtained within the same time frame as the circulatory parameters. Adults (age ≥ 20 years) who were admitted to the ICU following cardiovascular surgery between November 2024 and September 2025 were enrolled in the present study. The exclusion criteria were as follows: inability to obtain the outcome data on either postoperative day (POD) 2 or 3; use of mechanical ventilation (invasive positive pressure ventilation or non-invasive positive pressure ventilation); conditions affecting salivary secretion (e.g., Sjögren's syndrome, salivary gland tumor); oral bleeding; and refusal to participate in the study. The study followed the Standards for Reporting Diagnostic Accuracy Studies (STARD) 2015 recommendations [10]. The screening-to-analysis-set flow is illustrated in Figure 1.

We focused on postoperative cardiovascular surgical patients for the following reasons: in the early postoperative period, hypovolemia is more likely to become problematic due to excessive invasiveness; central venous catheter (CVC) placement is standard, providing continuous central venous pressure (CVP) measurement; and as part of the routine ICU cardiac ultrasonographic examination, the inferior vena cava (IVC) diameter and respiratory variation, venous excess ultrasound (VExUS) score, and left ventricular (LV) kissing sign can all obtained at the same time, allowing for strict reference criteria (composite criteria) without the need to perform additional new invasive procedures or tests for research purposes.

Outcomes

We prespecified the following hypothesis. For the diagnosis of hypovolemia using OMM as an index, the lower limit of the two-sided 95% confidence interval (CI) for both sensitivity and specificity, evaluated at the predefined tongue-dryness and Youden index-derived cutoff values, would be ≥0.60 (or ≥0.80 for either sensitivity or specificity). Additionally, the half-width of each 95% CI would be ≤0.12. Diagnostic accuracy was assessed using sensitivity, specificity, positive predictive value (PPV), negative predictive value (NPV), positive likelihood ratio (LR+), negative likelihood ratio (LR−), and accuracy, each calculated with two-sided 95% CIs. Given that PPV and NPV depend on prevalence, we also reported the cohort prevalence. The area under the curve (AUC) with 95% CIs from the receiver-operating characteristic (ROC) curve analysis was reported as a threshold-independent supplementary index. The primary outcome comprised sensitivity and specificity, with each of them being estimated with two-sided 95% CIs and evaluated at the two abovementioned prespecified cutoffs. For the secondary outcomes, we examined whether OMM was independently associated with hypovolemia and assessed its correlations with conventional indicators, including CVP, IVC diameter, VExUS score, and intraoperative water balance.

Index test and reference standard (timing and masking)

OMM as the index test utilized Mucus® (Appendix 1), also marketed as Moisture (approval number: 22200BZX00640000, Life Co., Ltd., Koshigaya, Saitama, Japan: hereafter, Mucus) [2-6]. Data acquisition was scheduled on the morning of POD 2; if same-day acquisition was not feasible, measurements obtained from POD 3 were used as backup. Cardiac ultrasonography was performed from 08:00 to 09:00 before ICU rounds, and OMM was measured from 09:00 to 10:00 after rounds. This acquisition schedule was prespecified and aligned with the routine ICU workflow at our institution to ensure feasibility and to standardize measurements within a fixed morning window. In addition, because staff shift handovers occur at 09:00 at our hospital, OMM measurements were scheduled after the handover to avoid the transition period and thereby minimize handoff-related variability in measurement conditions. The OММ measurements were performed by ICU-dedicated clinical engineers working in shifts, who had received prior technical guidance from dental hygienists. The measurement points were located from the central position, specifically 1 cm from the tip of the tongue, in a vertical direction [4,6]. Pressure intensity was fixed at approximately 200 g [2-4]. Three consecutive measurements were performed, with the median value adopted for analysis. To reduce the effects of tongue coating and other factors, the tongue was wiped with a moistened gauze before measurement, and the participant waited for 30 minutes before measurement. Inter-rater reliability was not formally assessed; however, the mucus measurement is a simple push-button procedure, and operator-related variability is expected to be minimal, further mitigated by using the median of three consecutive readings. To minimize acute influences on OMM, medication administration was avoided during the acquisition window, and water intake was avoided during the 30-minute waiting period after wiping and before OMM measurement. Additionally, because the measurement values can fluctuate by approximately 1%-2% depending on the differences in sensor covers [5], only manufacturer-approved covers were used for all devices. An indeterminate result was defined as a case in which no actual measured value was displayed in any of the three consecutive trials. Details of the measurement procedures are provided in Appendix 2. The reference standard for hypovolemia was a composite criterion predefined to combine CVP, IVC diameter, VExUS score [11], and cardiac ultrasound findings (described later).

The following variables were also collected: patients’ background characteristics (age, sex, body mass index (BMI), underlying diseases (diabetes, chronic respiratory failure (CRF), chronic heart failure (CHF), chronic kidney disease (CKD), collagen diseases, and so on), sepsis-related organ failure assessment (SOFA) [12], vital signs (heart rate, respiratory rate, systolic blood pressure), and hypovolemia-related indices (OMM, VExUS score [11], LV kissing sign (defined as the observation in which the ventricular walls are in close proximity/contact during systole in the parasternal short-axis view at the papillary muscle level) [13], maximum IVC diameter [14-16] and its respiratory variation [14,16] (measured in the subxiphoid long axis), and CVP [17]). Ultrasonographic examinations were performed each morning in the ICU as a routine procedure by a clinical laboratory technician, and data were retrospectively collected from the patients’ electronic medical records.

Furthermore, clinical engineers, dental hygienists, and clinical laboratory technologists were blinded to the study design and results. The detailed procedures, threshold definitions, and quality control are provided in Appendix 2.

Reference standard: rationale for a composite approach and justification for avoiding a fluid challenge

Fundamentally, the gold standard for assessing fluid responsiveness, an essential element of evaluating suspected hypovolemia, has been the fluid challenge [18,19]. However, in the present study, we deliberately avoided performing fluid challenges due to safety and ethical considerations. Our study population was limited to early ICU patients following cardiovascular surgery, and in this population, a positive fluid balance/fluid overload is associated with an increased risk of acute kidney injury and mortality [20,21]. Therefore, we substituted a quantitative composite using objective indices that are routinely obtained in the ICU (CVP, IVC, VExUS score, and LV kissing sign) within the same time frame, defined as “exclusion of congestion” plus “concordant findings suggestive of hypovolemia.” Although prior studies using OMM for hypovolemia (potentially encompassing free-water dehydration) assessment in emergency outpatients relied on the subjective judgment of physicians [2], the novelty of the present study lies in the use of a more scientific and quantitative composite reference in the ICU setting, where rich quantitative data are available. We defined hypovolemia as positive according to the composite criteria if all of the following conditions were met: VExUS = 0 points (ensuring absence of congestion) [11]; an abnormality in at least one of the conventional indices, including CVP of <4 mmHg [17,18] maximum IVC diameter of <10 mm [14-16], or positive IVC respiratory variability [14,16]; and presence of LV kissing sign [13]. The VExUS criteria and procedural flow are summarized in Appendix 2.

Statistical analysis

For the prespecified threshold, because no officially endorsed cutoff for dehydration/hypovolemia diagnosis exists for mucus [3-6], we derived the optimal cutoff using the Youden index (selecting the threshold nearest to the upper-left corner). We used this cutoff value as the prespecified within-cohort algorithm, and reported it in the primary analysis. We computed the AUC value (95% CI) obtained through the ROC curve analysis. At the selected threshold, we constructed a 2 × 2 contingency table to report sensitivity, specificity, PPV, NPV, LR+, LR−, positive test rate, prevalence, and overall accuracy, each accompanied by two-sided 95% CIs. The CIs for proportions were calculated using the Wilson method, for likelihood ratios using the log method, and for AUC using the DeLong method. As a sensitivity analysis, we conducted an additional analysis using a dryness level of <27.9% [3,5,6] as the classification threshold for tongue dryness. Moreover, the superiority or inferiority of the two thresholds was evaluated using McNemar’s test (with continuity correction or exact as needed) to compare the related proportions. For transparency, we also cross-tabulated the test positivity between the two cutoffs, irrespective of the reference standard.

Using both the Youden index and tongue-dryness diagnostic thresholds, we divided the studied population into the applicable and non-applicable groups; then, we analyzed the differences between the two groups for statistical significance. The normality of the continuous variables was assessed using the Shapiro-Wilk test. If normality was confirmed, the F-test was used to check for homoscedasticity, followed by Student’s or Welch’s t-test. The Mann-Whitney U test was applied if the data were not normally distributed. Fisher’s exact test was used for categorical variables. Furthermore, as a secondary analysis, variables found to be significant in the univariate analysis were forcibly entered along with age and severity (SOFA) into a multivariate logistic regression model. To determine the correlation of OMM with CVP, IVC diameter, VExUS score, and intraoperative water balance, Pearson’s product-moment correlation coefficient was utilized for normally distributed variables, and Spearman’s rank correlation coefficient for non-normally distributed or ordinal variables.

Any missing data relating to the primary outcomes or unresolved indeterminate values after re-measurement according to the predefined procedures were excluded from the main analysis. The frequency of these exclusions is presented in the Results section and Figure 1.

Statistical analyses were conducted using Easy R (EZR, RcmdrPlugin.EZR; Saitama Medical Center, Jichi Medical University, Saitama, Japan), version 1.51 [22], with a two-sided significance level set at 0.05.

The present study was approved by the Ethics Committee of Yuuai Medical Center (approval no. R06R027). The purpose of the study, its non-interventional nature, and data anonymization were explained to the study participants (or their proxies) to obtain informed consent. In cases where direct or proxy consent was unavoidably difficult, the participants were given the opportunity to opt out via a web notice. All procedures followed the policies approved by the ethics review board.

Sample size

A pilot ROC analysis on 20 consecutive patients for hypovolemia detection using OMM suggested a provisional cutoff of 26.1, with a sensitivity of 0.666 and a hypovolemia prevalence of 0.30. Given that the primary objective was to ensure the precision of the sensitivity estimate, we used EZR’s “sample size calculation to keep the CI of a single-group proportion within a certain width” [22,23] setting the two-sided 95% CI half-width to ±0.12 to determine the required number of hypovolemia cases (n = 60) [22]. In the absence of published incidence data for hypovolemia as defined by our composite reference standard, the internal pilot prevalence (0.30) was adopted in accordance with methodological guidance for diagnostic accuracy studies [23]. Consequently, the required overall sample size was N = 200 [22].

The present study aimed to assess the diagnostic accuracy and operational feasibility of OMM for detecting hypovolemia. To strengthen the design and allow comparison or combination with conventional postoperative fluid indicators, we restricted enrollment to patients with mandatory CVC placement after cardiovascular surgery. Given the anticipated difficulty in data acquisition under these constraints, we prospectively designated the present work as a pilot study to provide point estimates and variance for future confirmatory research. Accordingly, the analyses were performed even if the target sample size was not achieved.

## Results

Participants and baseline characteristics

During the study period, 85 eligible patients were enrolled consecutively; 28 were excluded. Ultrasound was not performed in 24/85 (28.2%) due to missed orders; in nine of these cases (37.5%), this coincided with non-adherence to the scheduled OMM measurement timing. Four patients (4.7%) declined measurement or withheld consent. No indeterminate OMM readings were recorded per predefined procedures. The final analysis included 57 cases (Figure 1), all with complete data. The estimated required sample size was 200, which was not reached. The assessor of the reference standard was blinded to the index test results, with no breach of blinding identified. In nine cases (15.8%), the data obtained from POD 2 were unavailable, and those from POD 3 were substituted.

The patients’ mean age was 66.9 ± 10.7 years, and 40 patients (70.2%) were male. The median SOFA score was 11.0 (9.0-15.0). The comorbidities were as follows: CHF, 20 (35.1%); CKD, 11 (19.3%); CRF, seven (12.3%); stroke, seven (12.3%); and endocrine disorders, one (1.8%). OMM was 27.4 (24.70-29.10). According to the composite criteria, hypovolemia was observed in 7/57 (12.3%) patients. In the univariate analysis based on the Youden index-derived OMM cutoff (applicable = test-positive for hypovolemia at ≤26.1; non-applicable = test-negative at >26.1), the BMI differed significantly between the applicable and non-applicable groups: 22.6 (19.6-23.7) vs 24.3 (21.8-26.1) kg/m². Details of the ROC analysis, including the AUC and the Youden index-derived cutoff for intravascular volume depletion (hypovolemia), are provided in Appendix 3. The same evaluation using the tongue-dryness diagnostic cutoff value (applicable = <27.9%; non-applicable = ≥27.9%) revealed no other significant differences in the patients’ background characteristics between the applicable and non-applicable groups (Table 1).

**Table 1:** Baseline characteristics and univariable analysis a: Fisher’s exact test (Categorical variables); b: Student’s t-test (Continuous variables and normally distributed, equal variance); c: Welch’s t-test (Continuous variables and normally distributed); d: Mann-Whitney U test (Continuous variables and non-normally distributed); *: Statistically significant (p < 0.05) Using the Youden index-derived cutoff (≤ 26.1), BMI was the only baseline characteristic that differed between groups: 24.3 (21.8-26.1) vs 22.6 (19.6-23.7). For outcomes, OMM values differed significantly under both classification schemes, the Youden index-derived cutoff (≤ 26.1) and the predefined tongue-dryness threshold (< 27.9%). SOFA: sepsis-related organ failure assessment; APACHE II: Acute Physiology and Chronic Health Evaluation-Ⅱ; BMI: body mass index; HR: heart failure; BT: body temperature; RR: respiratory rate; SBP: systolic blood pressure; CHF: chronic heart failure; CKD: chronic kidney disease; CRF: chronic respiratory failure; CVP: central venous pressure; IVC: inferior vena cava; LV: left ventricular; WB: water balance; VExUS: venous excess ultrasound; OMM: oral mucosal moisture (values); POD: postoperative day

		Diagnostic performance of OMM values					
		Cutoff by Youden index ≤ 26.1			Tongue dryness index < 27.9		
	Total	Not applicable	Applicable		Not applicable	Applicable	
	N=57 (100%)	n=34 (59.7%)	n=23 (40.4%)	P-value	n=24 (42.1%)	n=33 (57.9%)	P-value
Patient status							
Age (years)	66.9 ± 10.7	65.8 ± 11.5	68.6 ± 9.2	0.340^b^	65.8 ± 12.8	67.7 ± 8.9	0.503^b^
Sex (Male)	40 (70.2%)	24 (70.6%)	16 (69.6%)	>0.99 ^a^	17 (70.8%)	23 (69.7%)	>0.99 ^a^
SOFA (points)	5.0 (3.0-7.0)	4.0 (3.0-7.0)	5.0 (3.5-6.5)	0.322^d^	4.0 (3.0-7.0)	5.0 (3.0-7.0)	0.751 ^d^
BMI (kg/m^2^)	23.4 (20.9-25.1)	24.3 (21.8-26.1)	22.6 (19.6-23.7)	0.0428^d^^ *^	24.4 (21.7-25.6)	22.8 (20.6-24.4]	0.146^d^
Data acquired on POD 2	48 (84.2%)	29 (85.3%)	19 (82.6%)	>0.99 ^a^	19 (79.2%)	29 (87.9%)	0.47 ^a^
Vital sign							
- HR (/min)	74.9 ± 13.9	75.4 ± 14.3	74.1 ± 13.6	0.73^b^	74.9 ± 14.3	74.9 ± 13.8	0.998^b^
- BT (Celsius)	36.8 (36.6-37.3)	37.0 (36.6-37.5)	36.8 (36.6-37.1)	0.34 ^d^	36.9 (36.5-37.5)	36.8 (36.6-37.2)	0.668 ^d^
- RR (/min)	18.7 ± 5.28	18.0 (15.3-20.0)	19.0 (14.0-22.5)	0.922 ^d^	18.29 ± 4.0	18.97 ± 6.1	0.614^c^
- SBP (mmHg)	113.0 ± 17.5	114.5 ± 18.1	110.8 ± 16.6	0.444^b^	112.25 ± 19.0	113.55 ± 16.6	0.785^b^
Past medical history							
- CHF	20 (35.1%)	11 (32.4%)	9 (39.1%)	0.778 ^a^	7 (29.20%)	13 (39.4%)	0.575 ^a^
- CKD	11 (19.3%)	7 (20.6%)	4 (17.4%)	>0.99 ^a^	5 (20.8%)	6 (18.2%)	>0.99 ^a^
- CRF	7 (12.3%)	2 (5.9%)	5 (21.7%)	0.106 ^a^	1 (4.2%)	6 (18.2%)	0.220^a^
- Stroke	7 (12.3%)	2 (5.9%)	5 (21.7%)	0.106^a^	2 (8.3%)	5 (15.2%)	0.687^a^
- Endocrine diseases	1 (1.8%)	0 (0)	1 (4.3%)	0.404^a^	0 (0%)	1 (3.0%)	>0.99 ^a^
Alcohol drinker	26 (45.6%)	16 (47.1%)	10 (43.5%)	>0.99 ^a^	11 (45.8%)	15 (45.5%)	>0.99 ^a^
Smoker	22 (38.6%)	11 (32.4%)	11 (47.8%)	0.277 ^a^	8 (33.3%)	14 (42.4%)	0.586^a^
Volume parameter							
- CVP (mmHg)	10.0 (6.0-14.0)	10.0 (6.0-13.0)	11.0 (7.0-14.0)	0.568^d^	10.0 (7.8-13.3)	11.0 (6.0-14.0)	0.728^d^
- Max IVC size (mm)	18.4 ± 5.1	18.8 ± 4.2	17.8 ± 6.3	0.532^c^	19.3 ± 4.1	17.8 ± 5.7	0.278^b^
- IVC respiratory change	25 (43.9%)	12 (35.3%)	13 (56.5%)	0.174^a^	8 (33.3%)	17 (51.5%)	0.19^a^
- LV kissing	15 (26.3%)	8 (23.5%)	7 (30.4%)	0.76 ^a^	4 (16.7%)	11 (33.3%)	0.226^a^
- WB in operation (L)	1.84 (1.21-2.73)	1.82 (1.21-2.69)	1.93 (1.21-2.66)	0.916^d^	1.87 (1.21-3.77)	1.77 (1.21-2.34)	0.602^d^
- VExUS score (points)	0 (0-1)	0 (0-1.0)	0 (0-.5)	0.432^d^	0 (0-0)	0 (0-1)	0.125^d^
Outcome							
- OMM value (points)	27.4 (24.7-29.1)	28.9 (27.7-29.7)	24.0 (18.8-25.5)	< 0.00001^d *^	29.3 (28.8-30.1)	25.3 (22.8-26.3)	< 0.00001^d *^
- Intravascular volume depletion positive by composite criteria	7 (12.3%)	2 (5.9%)	5 (21.7%)	0.106 ^a^	1 (4.2%)	6 (18.2%)	0.22^a^

Missingness was primarily attributable to operational/workflow factors occurring before complete paired measurements were obtained; in particular, missed ultrasound orders reflected workflow/ordering failures. No participants were excluded based on OMM results. These exclusions were primarily operational rather than outcome-driven.

Primary diagnostic accuracy

Using the Youden index, the optimal threshold for hypovolemia was 26.1. The Youden-based cutoff derived from the ROC analysis is shown in Appendix 3. Sensitivity was 0.714 (95% CI, 0.290-0.963); specificity, 0.640 (95% CI, 0.492-0.771); AUC, 0.640 (95% CI, 0.476-0.804); PPV, 0.217 (95% CI, 0.075-0.437); NPV, 0.941 (95% CI, 0.803-0.993); LR+, 1.984 (95% CI, 1.092-3.604); and LR−, 0.446 (95% CI, 0.136-1.467). The positive test rate was 40.4% (23/57). Using the tongue-dryness cutoff value of <27.9%, sensitivity was 0.857 (95% CI, 0.421-0.996); specificity, 0.460 (95% CI, 0.318-0.607); PPV, 0.182 (95% CI, 0.070-0.355); NPV, 0.958 (95% CI, 0.789-0.999); LR+, 1.587 (95% CI, 1.068-2.359); and LR−, 0.311 (95% CI, 0.049-1.954). The positive test rate was 57.9% (33/57). Diagnostic accuracy was higher with the tongue-dryness cutoff: 0.509 (95% CI, 0.373-0.644) versus the Youden threshold: 0.649 (95% CI, 0.511-0.771); the difference was not significant (McNemar p > .99). The detailed results are provided in Table 2 and Appendix 4; cross-tabulations of each threshold against the composite reference are shown in Appendix 5.

**Table 2:** Comparison of diagnostic performance for intravascular dehydration (Youden threshold (≤26.1) vs tongue dryness threshold (<27.9)) Values are proportions (95% CI) unless noted. No formal hypothesis testing was performed for sensitivity, specificity, and positive/negative predictive value. The p-value shown refers only to the paired comparison of overall diagnostic accuracy using McNemar’s chi-squared test with continuity correction. There was no significant difference in diagnostic accuracy between the Youden index-derived cutoff (≤ 26.1) and the predefined tongue-dryness threshold (< 27.9%). AUC: area under the curve; 95% CI: 95% confidence interval; OMM: oral mucosal moisture (values); N/A: not applicable

	Cutoff by Youden index ≤ 26.1	Tongue dryness index < 27.9	McNemar’s chi-squared test (with continuity correction), P-value
AUC	0.640 (0.476-0.804)	Not applicable	N/A
Test positive rate	0.404 (0.276–0.542)	0.579 (0.441-0.709)	N/A
Prevalence	0.123 (0.051–0.237)	0.123 (0.051-0.237)	N/A
Sensitivity	0.714 (0.290–0.963)	0.857 (0.421-0.996)	N/A
Specificity	0.640 (0.492–0.771)	0.460 (0.318-0.607)	N/A
Positive predictive value	0.217 (0.075–0.437)	0.182 (0.070-0.355)	N/A
Negative predictive value	0.941 (0.803–0.993)	0.958 (0.789-0.999)	N/A
Diagnostic accuracy	0.649 (0.511–0.771)	0.509 (0.373-0.644)	>0.99
Positive likelihood ratio	1.984 (1.092–3.604)	1.587 (1.068-2.359)	N/A
Negative likelihood ratio	0.446 (0.136–1.467)	0.311 (0.049-1.954)	N/A

The distribution of positive classifications under the two thresholds is shown in Appendix 4. Given that the Youden threshold (≤26.1) is stricter than the tongue-dryness cutoff (<27.9%), all Youden-positive cases were a subset of the tongue-dryness-positive group. This cross-tabulation is descriptive and not conditioned on the reference standard; its purpose differs from that of the paired accuracy comparison.

Secondary and sensitivity analyses

In the multivariable logistic regression analysis, including age and SOFA (forced), along with BMI (significant in the univariable analysis) and OMM (variance inflation factor < 2 for all; model AUC value of 0.709 (95% CI, 0.567-0.85)), no independent predictor of hypovolemia was identified (all p > 0.05) (Table 3).

**Table 3:** Multivariable logistic regression for intravascular dehydration On multivariable logistic regression, no independent predictors of intravascular dehydration (hypovolemia) were identified. The AUC was 0.497 (95% CI, 0.345–0.649), indicating poor/low discrimination. SOFA: sepsis-related organ failure assessment; BMI: body mass index; OMM: oral mucosal moisture (values); OR: odds ratio; VIF: variance inflation factor; AUC: area under the receiver operating characteristic curve

	OR (95% CI)	P-value	VIF	AUC (95% CI)
Age (years)	1.01 (0.937-1.09)	0.776	1.028	0.709 (0.567-0.85)
SOFA (points)	1.16 (0.916-1.47)	0.217	1.079	
BMI (kg/m^2^)	0.979 (0.806-1.19)	0.826	1.090	
OMM (points)	1.00 (0.885-1.13)	0.977	1.005	

**Figure 2:**
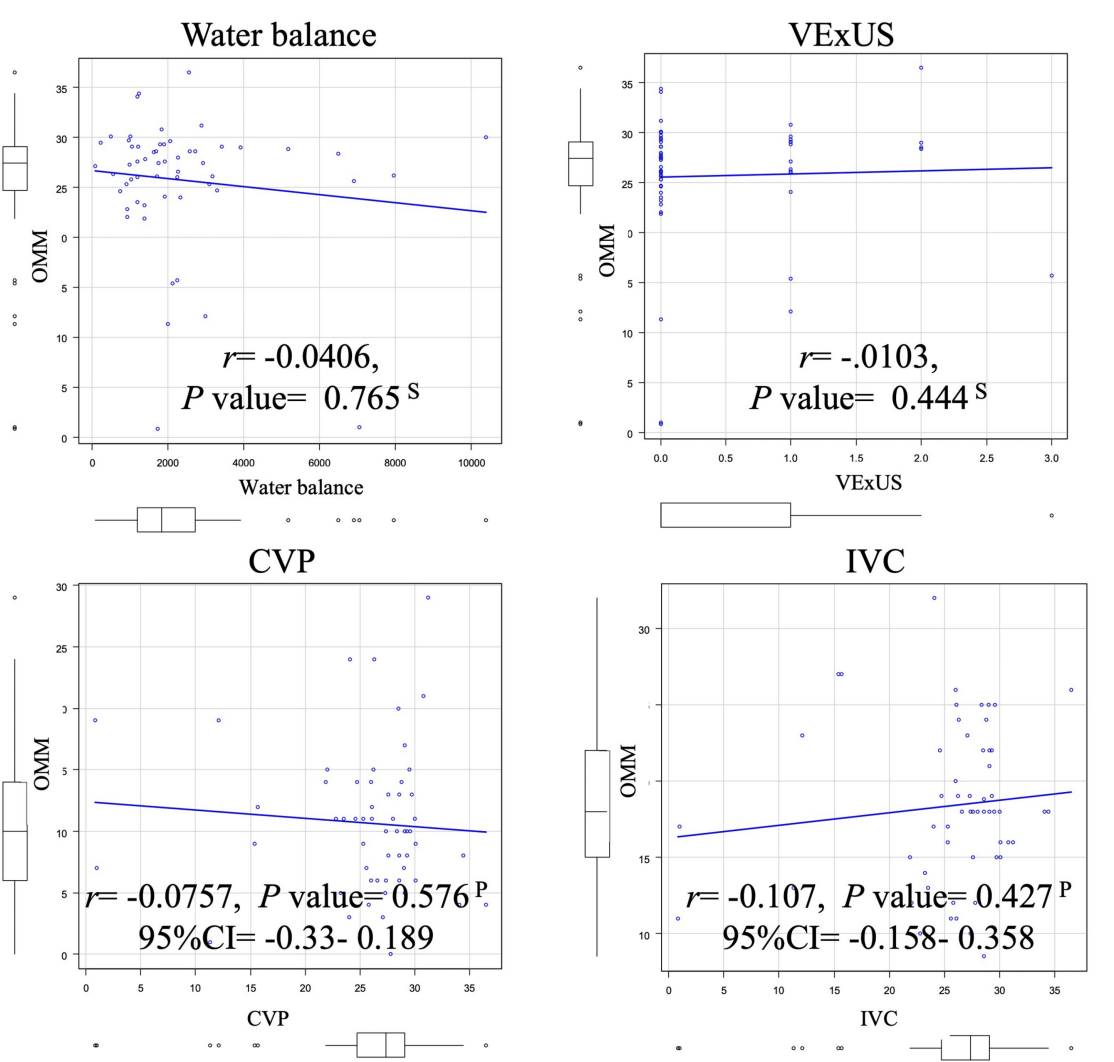
Correlation analyses No statistically significant correlations were observed between OMM and water balance, VExUS score, CVP, or IVC diameter/respiratory variation. P: Pearson; S: Spearman; OMM: oral mucosal moisture values; VExUS: venous excess ultrasound; CVP: central venous pressure; IVC: inferior vena cava; 95% CI: 95% confidence interval

In the correlation analyses, OMM was not significantly associated with CVP (r = −0.0757; 95% CI, −0.33 to 0.189; p = 0.576), maximum IVC diameter (r = −0.107; 95% CI, −0.158 to 0.358; p = 0.427), VExUS score (r = -0.0103; p = 0.444), or intraoperative water balance (r = −0.0406; p = 0.765) (Figure 2).

## Discussion

Primary outcome

This study should be interpreted as a pilot. Because enrollment and, in particular, the number of reference-standard positive cases were substantially lower than planned, estimates of sensitivity and specificity are imprecise with wide confidence intervals. For the data-driven threshold obtained by maximizing the Youden index (≤26.1) [24], sensitivity was 0.714 (95% CI, 0.290-0.963); specificity, 0.640 (95% CI, 0.492-0.771); PPV, 0.217 (95% CI, 0.075-0.437); NPV, 0.941 (95% CI, 0.803-0.993); accuracy, 0.649 (95% CI, 0.511-0.771); LR+, 1.984 (95% CI, 1.092-3.604); and LR-, 0.446 (95% CI, 0.136-1.467). The AUC value (auxiliary metric) was 0.640 (95% CI, 0.476-0.804). Although the point estimates for sensitivity and specificity met the rule-of-thumb target of ≥0.60, the wide CIs with lower bounds of <0.60 indicate a low diagnostic accuracy. Moreover, these findings could not exclude the possibility of optimism bias arising from optimizing and evaluating the cutoff in the same dataset [25]. At the prespecified tongue-dryness index cutoff (<27.9), the sensitivity was 0.857 (95% CI, 0.421-0.996), specificity, 0.460 (95% CI, 0.318-0.607); PPV, 0.182 (95% CI, 0.070-0.355); NPV, 0.958 (95% CI, 0.789-0.999); accuracy, 0.509 (95% CI, 0.373-0.644); LR+, 1.587 (95% CI, 1.068-2.359); and LR−, 0.311 (95% CI, 0.049-1.954). Only sensitivity exceeded 0.80 at the point estimate level, but the lower confidence bounds for both sensitivity and specificity remained at <0.60, undermining reliability. This AUC value is a threshold-independent auxiliary index and is not applied directly to an operational cut point [26]. Moreover, paired comparison of accuracy between thresholds showed no significant difference (McNemar p > .99).

Taken together, the cutoff Youden and tongue-dryness index values did not permit sufficiently precise estimation of diagnostic performance for hypovolemia, and our a priori hypothesis was not supported. Given the cohort prevalence of 0.123 (95% CI, 0.051-0.237), the consistently high NPVs (cutoff Youden index value: 0.941 (0.803-0.993); cutoff tongue-dryness index value: 0.958 (0.789-0.999)) suggest that OMM may help minimize the missed cases at low pretest probability when used as an adjunct alongside dynamic hemodynamic assessments. However, the low PPVs and modest LRs argue against using OMM as a stand-alone trigger for fluid administration in postoperative cardiac patients, where the risk of misclassification and fluid overload carries considerable clinical consequences [19,20].

We evaluated OMM against a prespecified composite reference in the postoperative cardiovascular surgical ICU, which required absence of congestion (VExUS score = 0) and concordant low-volume surrogates (CVP, IVC findings, and LV kissing sign). The negative findings in our research are physiologically coherent. Mucus quantifies moisture at the superficial tongue mucosa (extravascular) and was developed and validated for dry-mouth assessment [3-6]. Contrarily, in the postoperative cardiovascular ICU, endothelial injury and cardiopulmonary bypass promote capillary leak, extravascular sequestration of fluid, and tissue edema with relative intravascular hypovolemia [9,27]. This layer-target mismatch makes OMM unlikely to sensitively reflect intravascular volume depletion. Given that the VExUS assessment evaluates venous congestion rather than low-volume status, we required a VExUS score of 0 in the composite reference. This ensured that low-volume surrogates (e.g., CVP, IVC findings, and LV kissing sign) were interpreted as indicative of hypovolemia only in the absence of congestion [11]. This design choice reduces the risk of misclassifying patients with congestion as having hypovolemia in this postoperative cohort. A prior emergency department study reported a negative correlation between the dehydration level and OMM (r = −0.686); however, that study relied on a composite of laboratory tests and clinician judgment to grade dehydration, introducing subjectivity and potentially capturing free-water deficit in addition to hypovolemia [2]. Moreover, in older adults, subjective dry mouth demonstrates low sensitivity (0.08-0.30) and moderate specificity (0.67-0.88) for detecting water-loss dehydration; accordingly, it should be treated as a supplementary rather than a primary diagnostic sign [1]. Oral dryness has been cited as a sign of dehydration, but the literature often conflates dehydration (free-water deficit) with volume depletion/hypovolemia (intravascular volume loss), further complicating the interpretation across different settings [7,8]. These considerations support our finding that OMM should not be repurposed as a stand-alone screening test for intravascular volume depletion.

Secondary outcomes

In the multivariable analysis, including age and SOFA (forced), along with BMI (significant in the univariable analysis) and OMM, no independent predictor of hypovolemia emerged (Table 3). Moreover, the correlation analyses showed no meaningful associations between OMM and CVP, IVC diameter, VExUS score, or intraoperative water balance (Figure 2). Taken together, the absence of independent predictors in the multivariable model (Table 3) and the lack of correlation between OMM and reference measures, CVP, IVC diameter, VExUS score, and intraoperative water balance (Figure 2), align with the abovementioned physiological axis mismatch. Beyond this, several study features likely contributed to the modest discrimination. On the comparator side, the reference measures emphasize different physiological constructs already noted, which were as follows: CVP reflects a static pressure and is a poor surrogate for volume responsiveness or circulating volume [28]; a single-point IVC diameter is vulnerable to misclassification toward both under- and over-volume states [14,18]; and VExUS assessment evaluates venous congestion rather than low-volume status [11]. For intraoperative water balance, the discrepancies in measurement timing and perioperative fluid/ventilatory management likely introduce confounding. Considering these limitations and varying physiological mechanisms, OMM is unlikely to substitute for or meaningfully complement the evaluation of hypovolemia.

Generalizability

Our cohort comprised adults in a postoperative cardiovascular ICU who were not on mechanical ventilation, a population prone to relative hypovolemia after major surgical stress. Therefore, external validity primarily applies to similar postoperative ICU patients who are not on mechanical ventilation, and extrapolation beyond this patient group should be cautious. Within this clinical context, where capillary leak and perioperative care can uncouple tissue moisture from the circulating volume, OMM offered little incremental value and should not be used alone. Contrarily, performance in other populations, including general emergency department patients, community-dwelling older adults, non-postoperative ICU patients, or patients on mechanical ventilation, may differ; however, these contexts involve distinct physiological axes and assessment frameworks, and the available evidence is limited, so direct extrapolation is not appropriate [1,2,11]. Any consideration of OMM as a potential rule-out adjunct at low pretest probability is beyond the primary aims of the present study, and this remains hypothesis-generating; confirming this would require dedicated investigations in noncritical settings.

Limitations

The present single-center prospective observational study was limited to postoperative cardiovascular surgical patients in the ICU, making a spectrum effect likely and restricting external validity. For ethical and safety reasons, we avoided performing a fluid challenge as the gold standard and instead used composite criteria (VExUS = 0 with CVP, IVC findings, and LV kissing sign). Given that VExUS evaluates venous congestion rather than directly defining a low-volume status, residual misclassification cannot be excluded. Although index testing was standardized and mutually blinded, inter-rater reliability was not assessed, and measurements were obtained at one time point, precluding assessment of diurnal variability. Although assessments were standardized within a fixed morning window and intake/medication was avoided during the acquisition period, residual unmeasured time-varying factors in routine ICU care (e.g., transient oral conditions or ambient humidity) cannot be fully excluded. Any remaining ≤60-minute separation between ultrasonography-derived reference components and OMM measurement is therefore more likely to introduce random variability than systematic measurement bias. The relatively high rate of missed ultrasound orders (28.2%) reduced the number of complete paired assessments and may have contributed to imprecision of the diagnostic accuracy estimates. Moreover, the target sample size of 200 was not achieved (analyzed n = 57). This shortfall reflected a limited number of eligible patients and an operational requirement to limit measurements to clinical engineers to preserve uniformity, which constrained throughput. Because analyses were restricted to complete-case paired measurements, some selection bias related to clinical workload or patient factors (including refusal/withheld consent) cannot be fully excluded. As a result, the CIs were wider. The study was under-enrolled, and the number of hypovolemia cases was markedly smaller than planned. Consequently, diagnostic accuracy estimates had limited precision with wide confidence intervals, restricting confirmatory interpretation. To safeguard internal validity, we used mutual blinding, a prespecified acquisition window, predefined rules with sensitivity analyses, and STARD-compliant reporting. However, the possible optimism remains from selecting the Youden-based cutoff and evaluating it in the same dataset. Despite some optimism for improved performance, our results were negative. Additional enrollment was expected to narrow the CIs; however, it might not materially improve accuracy. Therefore, data collection was halted.

Future studies should prespecify an acceptable balance between the false negatives and false positives, establish the corresponding operating cutoff a priori, and validate it in a multicenter external study. Comparator selection should prioritize dynamic hemodynamic indices over static proxies alone to ensure the reference standard aligns with the physiological construct of hypovolemia.

## Conclusions

In this single-center postoperative cardiac ICU cohort study, OMM showed no association with intravascular volume and demonstrated only limited discrimination. OMM is, therefore, not supported as a stand-alone screening index for hypovolemia in critically ill ICU patients; if used, it should be limited to a supplemental measure alongside dynamic hemodynamic indices, not used as a trigger for fluid administration. These data relate specifically to intravascular depletion and do not rule out the potential utility of OMM for detecting free-water dehydration in other settings. The assumption “dry mouth = dehydration/hypovolemia” should be interpreted in light of population, pathophysiology, and reference standards.

## Figures and Tables

**Figure 3:**
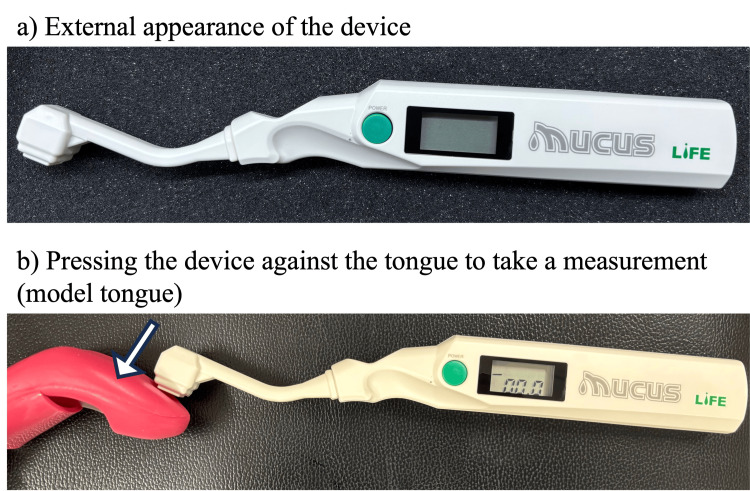
Mucus® (actual product photographs, overview and tongue placement) Press the device tip perpendicularly against the tongue to measure at the midline, 1 cm posterior to the tongue tip, applying a fixed pressure of ~200 g. During actual measurements, a dedicated disposable cover is fitted to the device tip.

**Figure 4:**
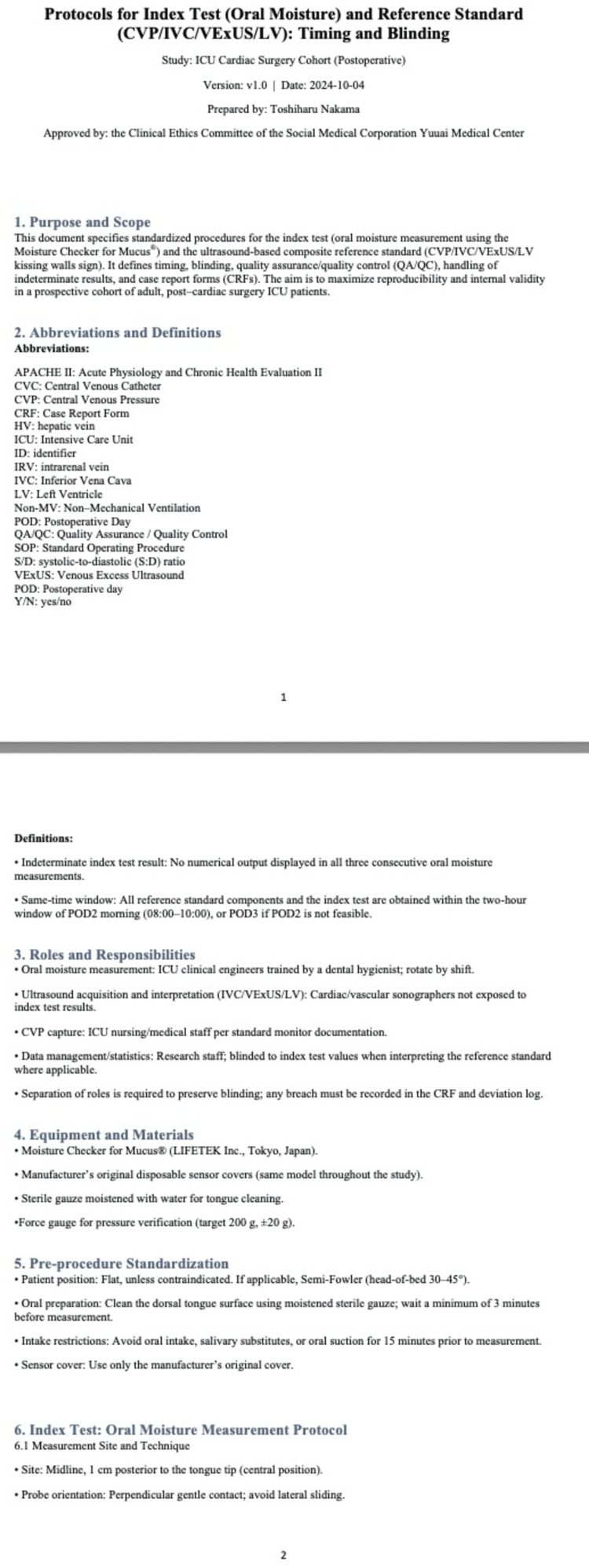
Protocols for index test (oral moisture) and reference standard (CVP/IVC/VExUS/LV) (timing and blinding) (part 1) Protocols for the index test, oral mucosal moisture, and the reference standard (CVP, IVC, VExUS, LV), summarizing measurement windows and blinding procedures. All abbreviations are defined in the manuscript.

**Figure 5:**
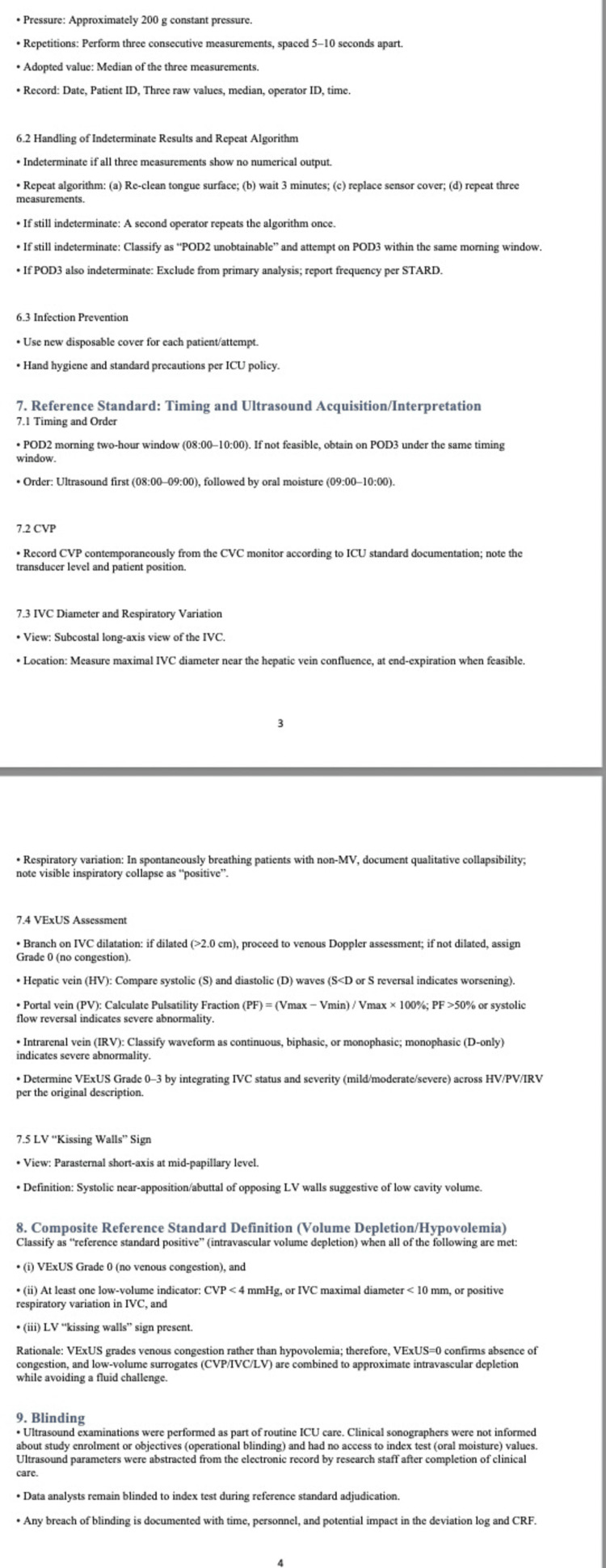
Protocols for index test (oral moisture) and reference standard (CVP/IVC/VExUS/LV) (timing and blinding) (part 2) Protocols for the index test, oral mucosal moisture, and the reference standard (CVP, IVC, VExUS, LV), summarizing measurement windows and blinding procedures. All abbreviations are defined in the manuscript.

**Figure 6:**
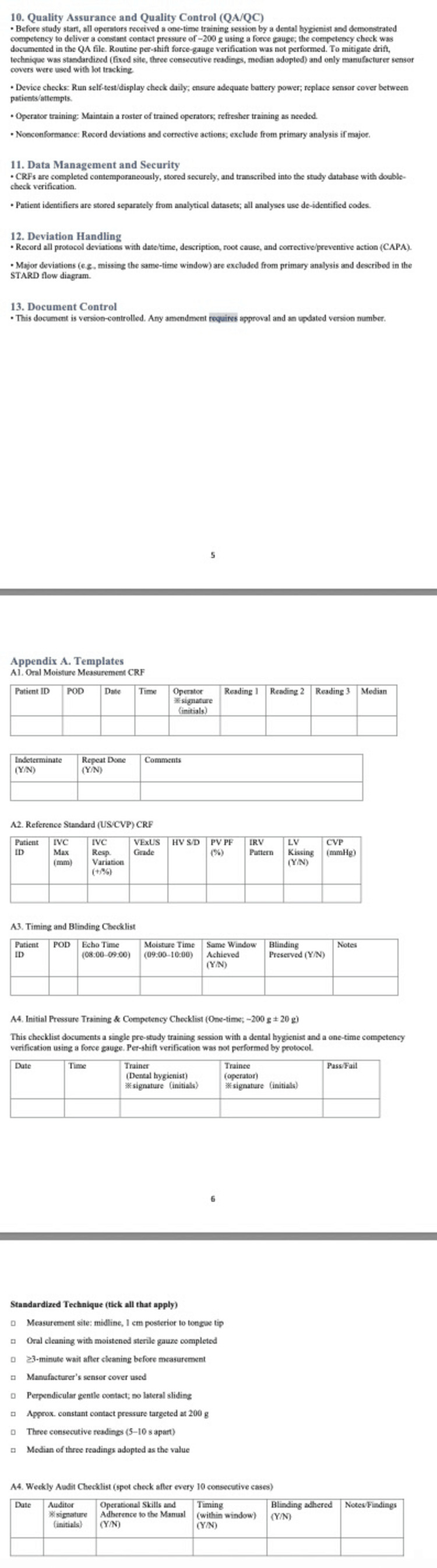
Protocols for index test (oral moisture) and reference standard (CVP/IVC/VExUS/LV) (timing and blinding) (part 3) Protocols for the index test, oral mucosal moisture, and the reference standard (CVP, IVC, VExUS, LV), summarizing measurement windows and blinding procedures. All abbreviations are defined in the manuscript.

**Figure 7:**
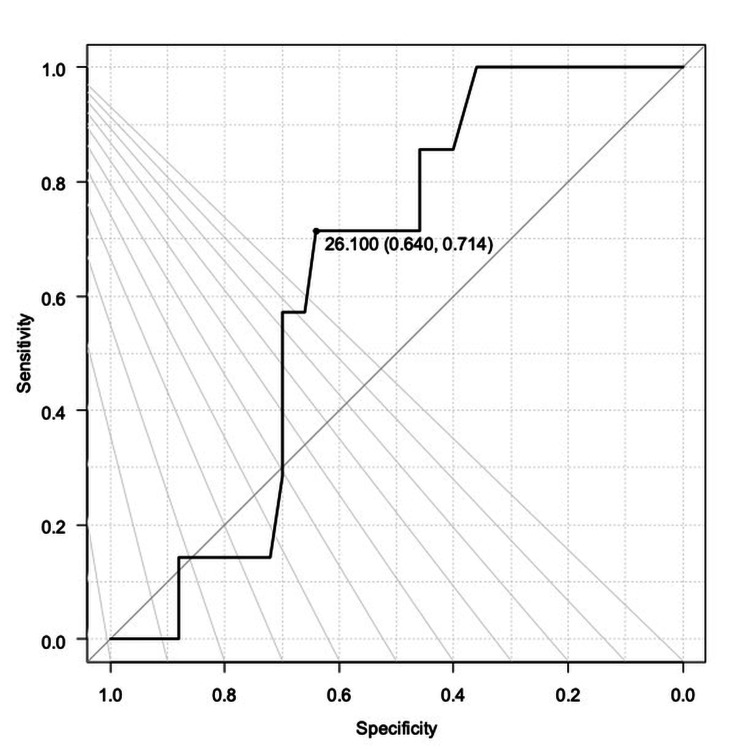
Cutoff value based on the Youden index for intravascular volume depletion calculated from the AUC in ROC analysis Threshold: 26.10, specificity: 0.64, sensitivity: 0.7142857, AUC: 0.64, 95% CI: 0.476 - 0.804. ROC: receiver operating characteristic; AUC: area under the curve; 95% CI: 95% confidence interval

## Appendices

Appendix 1 

Appendix 2

Appendix 3

Appendix 4

**Table 4:** Paired cross-classification of thresholds (n=57) This table cross-classifies the two thresholds without conditioning on the reference standard. Marginal proportions: Youden positive 23/57 (0.404), Mucus positive 33/57 (0.579).

	Youden + (≤26.1)	Youden − (>26.1)	Row total
Tongue dryness index + (<27.9)	23	10	33
Tongue dryness index − (≥27.9)	0	24	24
Column total	23	34	57

Appendix 5

**Table 5:** Cross-tabulation (Youden ≤26.1 and tongue dryness <27.9) (n=57) This table cross-tabulates each threshold against the composite reference (n = 57), reporting TP/FP/FN/TN counts and row/column margins. Because the Youden cutoff (≤ 26.1) is stricter than tongue-dryness < 27.9%, all Youden-positive cases fall within the tongue-dryness-positive group. The table is descriptive and not a paired comparison; it is not intended to test differences in accuracy (for which McNemar’s test was used). TP: true positive; FP: false positive; FN: false negative; TN: true negative

	Youden + (≤26.1)	Youden − (>26.1)	Row total	Tongue dryness + (<27.9)	Tongue dryness − (≥27.9)	Row total
Composite + (ref +)	5 (TP)	2 (FN)	7	6 (TP)	1 (FN)	7
Composite − (ref −)	18 (FP)	32 (TN)	50	27 (FP)	23 (TN)	50
Column total	23	34	57	33	24	57
